# General Intelligence in Another Primate: Individual Differences across Cognitive Task Performance in a New World Monkey (*Saguinus oedipus*)

**DOI:** 10.1371/journal.pone.0005883

**Published:** 2009-06-17

**Authors:** Konika Banerjee, Christopher F. Chabris, Valen E. Johnson, James J. Lee, Fritz Tsao, Marc D. Hauser

**Affiliations:** 1 Department of Psychology, Harvard University, Cambridge, Massachusetts, United States of America; 2 Department of Psychology, Union College, Schenectady, New York, United States of America; 3 Department of Biostatistics, University of Texas M.D. Anderson Cancer Center, Houston, Texas, United States of America; 4 Department of Applied Mathematics, University of Texas M.D. Anderson Cancer Center, Houston, Texas, United States of America; 5 Department of Human Evolutionary Biology, Harvard University, Cambridge, Massachusetts, United States of America; Università di Parma, Italy

## Abstract

**Background:**

Individual differences in human cognitive abilities show consistently positive correlations across diverse domains, providing the basis for the trait of “general intelligence” (*g*). At present, little is known about the evolution of *g,* in part because most comparative studies focus on rodents or on differences across higher-level taxa. What is needed, therefore, are experiments targeting nonhuman primates, focusing on individual differences within a single species, using a broad battery of tasks. To this end, we administered a large battery of tasks, representing a broad range of cognitive domains, to a population of captive cotton-top tamarin monkeys (*Saguinus oedipus*).

**Methodology and Results:**

Using a Bayesian latent variable model, we show that the pattern of correlations among tasks is consistent with the existence of a general factor accounting for a small but significant proportion of the variance in each task (the lower bounds of 95% Bayesian credibility intervals for correlations between *g* and task performance all exceed 0.12).

**Conclusion:**

Individual differences in cognitive abilities within at least one other primate species can be characterized by a general intelligence factor, supporting the hypothesis that important aspects of human cognitive function most likely evolved from ancient neural substrates.

## Introduction

Whenever humans are presented with cognitive tasks where a correct response can be objectively determined, their performances are almost always positively correlated [1], [2]. This consistent finding implies a “general intelligence” (*g*) factor, a capacity that unifies or underpins all human cognitive abilities [3], [4]. Scores on *g*-loaded ability tests show substantial genetic influence [5] and a diverse array of physiological correlates that include overall brain volume [6], [7]. This is intriguing because brain size is known to have increased substantially over the evolution of the primate lineage leading to *Homo sapiens sapiens* [8].

If there are indeed evolutionary homologues of the mechanisms subserving the *g* factor in humans, then sufficient extant genetic variation should lead to similar factors underlying performance on cognitive tasks in other primates. A recent meta-analysis of several primate genera provides support for this prediction [9]. Further evidence is required, however, before the general factor among genera can be attributed to causal sources similar to those underlying human *g*. In particular, it is presently unclear whether the positive correlations found across primate species arise because of an intrinsic dependence on a set of common mechanisms—as in humans [10], [11]—or because of spurious sources of association.

What is necessary, therefore, is a detailed study of within-species variation. To date, the most substantial evidence for a general factor in animals comes from laboratory studies of mice and rats [12]–[18]. Studies of primates are either limited because of the narrow range of tasks within the test battery or because of contrasts between taxa. The primary goal of our study was to explore the possibility of a homologue to general intelligence in a nonhuman primate species using a broad task battery. To this end, we tested a sample of 22 cotton-top tamarin monkeys (*Saguinus oedipus*) on 11 tasks covering a wide range of cognitive domains. Our battery included the following tasks: occluded reach, targeted reach, A-not-B, reversal learning, exploration, numerical discrimination, acoustic discrimination, object tracking, social tracking, hidden reward retrieval, and a food extraction puzzle. Using a Bayesian latent variable analysis, we provide evidence of a general factor underlying individual differences in cognitive abilities within this primate species.

## Results

Because it was not possible to quantitatively asses the cognitive performance of non-human primates across a broad range of tasks using fine-grained numerical scales, we represented the data collected during our experiment in the form of ranks.. Given our use of ranked observations, standard repeated measures analyses were not appropriate. We therefore analyzed our data using a Bayesian latent variable model that was previously used for inter-species analysis of non-human primate cognitive data and has been vetted in the mainstream statistical literature [9], [19]. Using this model framework, we first estimated the loading of each task on a group factor (reflecting a particular cognitive domain or function) and on a top-level general factor analogous to the human *g* factor. The posterior expectations of the task-specific variances ranged from 1.00 to 1.35, while the posterior expectations of the group factors' variances ranged from 0.012 to 0.045. It is clear that a much greater proportion of the variance in the posited latent variable underlying any given task is attributable to task-specific sources such as experimental error rather than to a group factor. This implies that the correlational structure of the tasks can be accounted for by a general factor alone. For this reason we re-estimated the model without the group factors. Checks of model adequacy indicated sufficient fit (Supplemental Figures S1 and S2).

**Figure 1:**
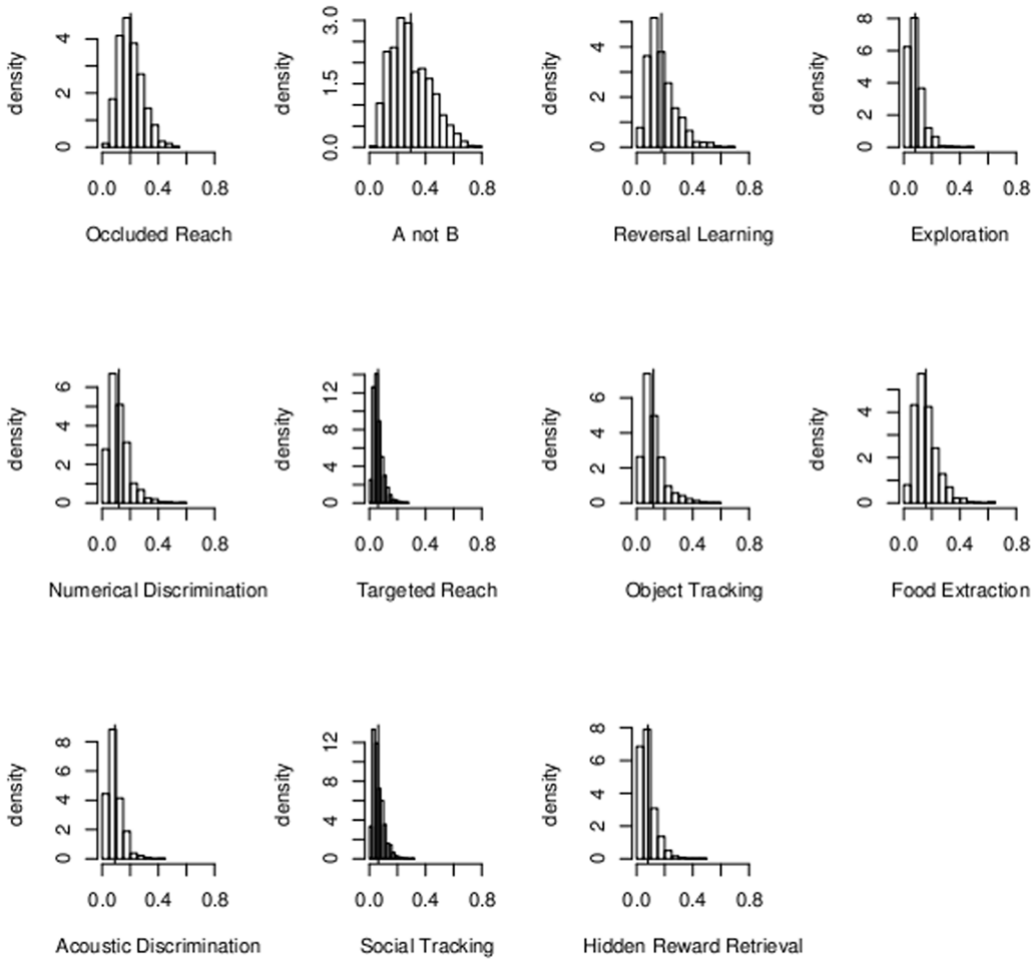
Posterior histograms of Var(*θ*)/[(1/*γ_j_*) + Var(*θ*)]. The proportion of variance in the latent variable underlying the *j*th task attributable to the general factor. Each panel displays the marginal posterior histogram of this proportion. The square root of the proportion is called the “loading” of the task on the general factor—the correlation between the task and the general factor.

We next computed the marginal posterior distribution of the proportion of latent variance underlying each task attributable to the general factor. The square root of this proportion gives the “*g*-loading” of the task, which can be interpreted as the correlation between performance on the task and the general factor. Several features are evident from the posterior histograms of the proportions of the task variances attributable to the general factor (Figure 1). Critically, the modal estimate of every task's loading on the general factor is always positive (Table 1). This finding is of course consistent with the presence of a genuine general factor. However, although the 95% credibility intervals always exclude zero, the loadings are typically quite small, especially relative to what is usually observed in human cognitive test batteries [2], [6].

**Table 1:** Estimated Loadings of Cognitive Tasks on General Factor (Bayesian 95% credibility intervals in parentheses).

task	loading on general factor
A-not-B	.542 (.287, .775)
occluded reach	.449 (.268, .623)
reversal learning	.421 (.206, .595)
food extraction puzzle	.397 (.206, .595)
object tracking	.346 (.163, .589)
numerical discrimination	.346 (.167, .561)
acoustic discrimination	.302 (.152, .483)
exploration	.286 (.133, .473)
hidden reward retrieval	.285 (.128, .473)
social tracking	.253 (.121, .404)
targeted reach	.249 (.127, .392)

Interestingly, three of the four tasks with the lowest loadings (targeted reach, social tracking, exploration) are the ones with arguably the weakest claims to membership in the domain of cognitive abilities. This domain can be defined as those items or tests that tax a *mental ability* and to which responses can be unambiguously scored from *very wrong* to *very right* [1], [4]. This domain description can in turn be clarified by defining a “right” response as a decision made “on some grounds of truth: correspondence to reality or soundness of inference” [20] (p. 61). While the three aforementioned tasks were included in the battery based on prior research suggesting that they were correlated with cognitive ability, high performance scores on these tasks do not entail evidence of computations modeling reality. For example, it cannot be either right or wrong for an animal to spend any particular amount of time in the various parts of the box in the exploration task. In this light it is also notable that the tasks with the highest *g* loadings, including A-not-B and numerical discrimination, clearly fall within the cognitive domain, tapping capacities such as inhibitory control and number representation.

For each task we computed the posterior probabilities that it has a higher *g* loading than any other task, and the separation of the most and least *g*-loaded tasks is fairly clear (Supplemental Table S1). However, it may be that the pattern of loadings is partly attributable to differences across tasks in reliability. An examination of those tasks with replications suggests potentially significant differences in reliability (Supplemental Table S2).

64.5% of the pairwise comparisons in the observed data that did not result in ties were correctly predicted by the posterior expectations of the *θi*. The significance of this result can be tested under a frequentist approach. For each entry of the raw data matrix that is not missing, we drew a random sample from a standard normal distribution. The estimate of each subject's ability from this pseudo-data was taken to be the mean of its random draws. The random draws within each task were then adjusted to reflect the structure of ties in the real data. For instance, if a task yielded a four-way tie for first, the scores of the subjects ranked two through four on this task in the pseudo-data were set equal to the score of the top-ranked subject. We counted the proportion of pairwise comparisons in the pseudo-data not resulting in ties that were correctly predicted by the pseudo-estimates of ability.

In 10,000 replicates of this simulation, the proportion of replicates yielding a prediction rate exceeding that obtained in the actual data using the posterior expectations of the *θi* was only 0.0236. Note that we have not shown that ordering the subjects by their pseudo-data means is optimal for purposes of retaining the null hypothesis that the prediction rate reflects mere capitalization on chance. Nevertheless we take the low empirical *p*-value from our simulations as converging evidence that the posterior distributions of the *θ_i_* reflect the genuine structure in the data that is captured by our model of a general factor.

## Discussion

In this study we applied a Bayesian latent variable model of rank data to the results of a battery of 11 cognitive tasks administered to 22 cotton-top tamarins. These tasks are posited to measure a diversity of cognitive processes and content domains, including executive control, memory, attention, and problem solving, in social and non-social situations, with food and non-food motivators. The results provide evidence of individual differences in a general factor (*g*) of cognitive ability in cotton-top tamarins. The magnitude of the *g* factor's influence appears to increase as the task engages more systematic cognitive control, although the limitations of our data preclude certainty on this point.

One significant contrast with patterns typical of human data is an absence of compelling evidence for any group factors. A similar result emerged in the meta-analysis of primate genera conducted by Deaner et al. [9]. At present, we cannot offer a clear explanation for this disparity. One possibility is that experimental noise and task-specific sources of variance in measurements of nonhuman primates are substantially increased relative to their typical levels in human testing. The few tasks with replications do suggest that some tasks have rather low reliabilities (Supplemental Table S2). As a result, the contribution of group factors may be more difficult to discern in animal data. Note that this possibility also complicates any attempts to draw inferences from the fact that the estimated loadings of our tasks on their general factor are much smaller than typical *g-*loadings of ability tests taken by humans [4]. In future studies of this kind, we recommend the use of larger samples with more complete data sets per subject, thus leading to narrower credibility intervals. Further development of the Bayesian latent variable model used in this study may also allow replications of tasks to separate measurement error and task-specific variance. Such measures may shed light on the reasons for the relatively low loadings on the general factor observed in this study. If the smaller loadings turn out not to be solely attributable to measurement error, it may be that one trend in the evolutionary lineage leading to our species has been an increasing reliance on interfaces among distinct cognitive processors, some domain-general (e.g., memory, attention, executive control), and others domain-specific (e.g., object knowledge, mental state attribution).

It is sometimes argued that the presence of a general cognitive factor is inconsistent with the hypothesis that the human mind consists of highly specialized, domain-specific modules [23]. We reject this argument, appealing to a distinction between the qualitative architecture of cognition and the structure of quantitative differences [6], [24]. The explanations for the phenomena arising within these two spheres may reside in different levels of the reductionist hierarchy. For example, variation in low-level global properties of the neural substrate may induce a general factor underlying quantitative differences in high-level modules with distinct functions and operating principles. Indeed, discriminations making explicit use of number in the numerical discrimination task depend on a system for precise counts of distinct objects that is distinguishable from another system for the approximate representation of large magnitudes [25]. Further, systems that are specialized for solving one problem can be either constrained or enhanced by interfaces with other systems, either domain-specific or domain-general. For example, though the recursive operations that underpin language are virtually limitless (generating discrete infinity with respect to the number of meaningful expressions), they are constrained in comprehension and production by our domain-general working memory system. In sum, we argue that both architectural features and quantitative differences are worthy topics of investigation for evolutionarily-oriented scientists interested in phylogenetic patterns and adaptive functions, as well as cognitive scientists interested in the mechanisms that support these capacities.

One goal of our study was to secure evidence supporting the intrinsic nature of the general factor found in comparisons of different primate genera. As the presence of a general factor within a primate species indicated by a similar number and diversity of tasks indeed strengthens this supposition, our study has been successful in this regard. However, our pairwise prediction rate of 64.5%, although significantly greater than expected by chance, is smaller than the 85% found in the meta-analysis of primate genera [19]. Many of our tasks were similar to those considered in this meta-analysis. If we assume some similarity between the general factors found in the two studies and comparability of such relevant features as the degree of experimental noise, then the lower prediction rate in the tamarin comparisons suggests that variation among taxa in the general factor is large relative to variation within this species. Substantial variation among taxa is consistent with an important role for differentiation along this dimension in the evolution of the primate order.

Further research is necessary regarding the relationships among the respective general factors within different primate species (including *Homo sapiens*) and the general factor across primate taxa. In particular, we need a much more detailed analysis of the causal or correlational nature of the different neural and psychological processes that facilitate or constrain task performance. There is a significant positive correlation between the primate general factor and brain volume [26], a pattern observed within humans as well [27]. Future studies might seek to establish similar relationships between the replicated physiological correlates of the human *g* factor (brain size, white matter connectivity, levels of the neural metabolite N-acetylaspartate; for a review see [6]) and primate general factors at varying taxonomic levels. Of additional interest will be the connectivity between different domain-specific systems (e.g., capacity to understand others' mental states and abilities for numerical quantification in the context of cooperative games) and their links to more domain-general processes (e.g., memory for prior interactions and participants).

We acknowledge the great practical difficulties posed by a research program seeking to find such relationships within different primate species. There are, however, indications in the literature that it can be accomplished [28]. A promising place to begin is with species that are abundantly available in captivity, be they in research sites or zoos. For example, zoos and research labs throughout the United States have access to chimpanzees, rhesus monkeys and squirrel monkeys, three species representing each of the primary taxonomic groups (i.e., apes, old world monkeys, and new world monkeys). Such a research program is worthwhile because of the great theoretical interest that would attach to any positive results. In particular, we think of intelligence as a hallmark of the human species. But the mechanisms and representations that enter into human intelligence are unclear, as are the paths leading to its evolution. By specifying these ingredients, including the relevance of both domain-specific (e.g., language, number, theory of mind) as well as domain-general (e.g., inhibitory control, recursive computation, and attention) processes, we will be in a stronger position to guide future research into the cognitive evolution of our species.

## Materials and Methods

### Ethics Statement

All tasks conformed to the animal subject regulatory standards enforced by the Institutional Animal Care and Use Committee (IACUC) at Harvard University. The IACUC protocol number is 92-16, approved on 6/30/08. The welfare of the animals conformed to the requirements of the National Institute of Mental Health (NIMH). All animals were housed in cages exceeding the sizes stipulated in said requirements, together with conspecifics in their natural group compositions. All animals were given access to a rich diet of foods and engagement with a variety of psychologically enriching tasks. No animal was physically harmed or deliberately exposed to potential infection.

### General Procedure

We tested 22 adult cotton-top tamarin monkeys (10 females and 12 males) of mixed experimental history. Subjects ranged in age from 3 to 17 years. All subjects were housed in the same colony room in the Cognitive Evolution Laboratory at Harvard University. In addition to food given in experiments, all subjects were fed a nightly meal and maintained at approximately 10% less than their free-feeding weights in captivity; thus, we maintained subjects at a weight that more closely approximated those observed among wild subjects (400–465 g).

The battery consisted of 11 tasks that were administered between March 2007 and December 2007. Tasks were always administered between the hours of 8 am and 5 pm. Data were not collected on days where we anticipated any exceptional cause for excitement in the tamarin colony, such as a veterinary check or lab construction.

All experimenters were required to practice the task procedures together until attaining a high degree of uniformity in administration. Prior to each task, subjects voluntarily moved from their home cage into a transport cage and were then transferred to the appropriate testing room. By restricting testing to animals that voluntarily left their home cage, we provided more consistency with respect to motivational state.

Although we attempted to test all 22 subjects on each task, this was not possible due to several uncontrollable factors, including routine medical care, pregnancy, and occasional unwillingness to voluntarily enter the transport cage on the day of testing.

### Task Descriptions

The following provides a synopsis of each task within our battery; more details are given in Supplemental Protocol S1. Descriptive statistics are provided in Supplemental Table S3.

#### Occluded reach

The subject watched as the experimenter placed a food item (a quarter of a piece of Froot Loop cereal) in one of three positions behind a transparent Plexiglas barrier positioned in front of the subject's cage. In the *right* and *left* conditions, the experimenter placed the food at the edge of either side of the Plexiglas barrier; half of the food was positioned behind the barrier, and the other half was exposed to the subject. In the *center* condition, the food was located directly behind the center of the transparent barrier. The subject participated in five sessions of this task consisting of twelve trials each over five consecutive days. Each food position condition was presented four times within a session, and condition order was counterbalanced across all twelve trials. The percentage of *center* trials in which the subject successfully reached around the barrier (i.e., inhibited reaching straight for and into the barrier) to access the food was recorded for each session.

#### Targeted reach

A cable-tie glued to the top of the subject's transport cage was loaded with half of a raisin. The cable-tie and raisin were raised to a 60-degree angle by the side of the subject's cage, released, and allowed to oscillate in front of the subject's cage door. The subject was free to reach its hands or mouth through a small square hole in the transport cage door in order to grasp the swinging raisin. This procedure was repeated for five trials in a single session. The time required for the subject to successfully grasp the raisin was measured for each trial. Similar studies of rhesus macaques indicate that this type of task co-activates visual motion processing and motor command responses in the cerebral cortex [29].

#### A-not-B

Two opaque barriers were positioned in front of the subject's transport cage. Froot Loop quarters were placed behind barrier A on five consecutive trials. On the sixth trial, the subject watched the experimenter place the food behind barrier B. We recorded whether, on this sixth trial, subject reached behind the incorrect barrier A or the correct barrier B.

#### Reversal learning

The experimenter wore a red glove on one hand and a green glove on the other. Subjects were taught to associate a food reward (a quarter of a Froot Loop) with either the red glove or the green glove. In the test phase, the food reward was presented to the subject as in the training phase, except that the reward was concealed in the hand bearing the opposite-colored glove. In order to pass the test phase, the subject was required to choose correctly on ten out of the twelve trials in one session. The number of sessions required to pass the test phase were recorded.

#### Exploration

On consecutive days, subjects were taken to a large, open-field box to participate in seven different task conditions. Each subject was allowed to move about freely inside the box for five minutes. In five of the seven conditions, a different stimulus was located in the center of the box. In the two baseline conditions, no stimulus was present inside the box. Several dependent measures were examined as indicators of the subject's overall exploratory behavior and novelty preference. These dependent measures included time spent moving (versus stationary), time spent in physical contact with the stimulus, and time spent in each quadrant of the box. This task is similar to one that has been found to be correlated with a general cognitive factor in studies of mice [15], [30]. In humans, preference for novel objects in infancy has been found to predict IQ at later ages [31].

#### Numerical discrimination

Subjects watched as the experimenter loaded two clear petri dishes with different quantities of food items (quarters of Froot Loops). The following contrasts were used: 1 v. 2, 1 v. 3, 1 v. 4, 1 v. 5, 2 v. 3, 2 v. 4, 3 v. 4, and 4 v. 5. The subject was then allowed to choose one of the petri dishes by reaching a hand or mouth through one of two small holes in the transport cage. The subject was run in three sessions of this task consisting of ten trials each over three consecutive days. The proportion of trials in which the subject chose the larger number of food items was recorded. Variants of this task have been administered in previous studies assessing the cue (volume, density, or number) by which tamarins, marmosets, and rhesus macaques discriminate between two quantities of food [32], [33].

#### Acoustic discrimination

Subjects' rates of habituation to four biologically meaningful acoustic stimuli were measured. The four stimuli consisted of a tamarin alarm call, a goshawk alarm call, and the contact calls of two members of the lab colony known to the subjects. Stimuli were played when the subject was looking away from the speaker in order to allow for a maximal orientation response. A response was defined as orienting up and toward the speaker located behind the transport cage during the stimulus exposure or within two seconds of its termination. The subject was scored as habituated to the stimulus when it failed to orient toward the speaker for three consecutive stimulus exposures. The number of exposures required to habituate to each of the four stimulus conditions was measured; fewer needed exposures indicated better performance. A nearly identical protocol was used in a previous study of tamarins' patterns of habituation to the contact calls of familiar conspecifics [34]. Rapidity of habituation by human infants to previously presented stimuli is surprisingly predictive of their IQs measured at much later ages [35].

#### Object tracking

We measured the time that a subject spent tracking each of two different stimuli: a raisin (food object) and a metal screw (non-food object). The subject was exposed to both stimulus conditions once within a single session. The experimenter presented each stimulus for two seconds at a distance of 5 cm from the subject's cage door and then moved the stimulus in a fixed pattern comprised of straight lines, diagonal lines, and figure-8s. The percentage of the presentation time that the subject spent looking at the moving stimulus was measured for each condition. General attentional processes have been shown to be an important factor in tamarins' rate of learning in operant conditioning tasks [36].

#### Social tracking

Subjects were positioned inside a transport cage that was adjacent to a second transport cage containing a “stooge” 'animal. The transport cages were divided by an opaque barrier preventing the two animals from viewing each other except through four peepholes located in each corner of the barrier. The subject was allowed to watch the stooge through the peepholes while the stooge was foraging for pieces of Froot Loop cereal in a woodchip-filled trough attached to the side of the stooge’s cage. Though the subject could easily view the stooge, the positioning of the adjacent cages largely prevented the subject from viewing the trough. As a result the majority of the subject’s time spent looking through the peepholes involved tracking the stooge rather than the food stimulus. The subject was allowed to look through the peepholes during a single 60-second trial. The total time that the subject spent tracking the stooge through any of the four peepholes was measured.

#### Hidden reward retrieval

Subjects watched an experimenter bury a quarter of a Froot Loop in one of two food wells filled with woodchips. In each condition a different delay length was imposed before the food wells were pushed against the subject’s transport cage. The subject was exposed to seven delay conditions on consecutive days in the following order: no delay, 5-second delay, 10-second delay, 15-second delay, 20-second delay, 25-second delay, and 30-second delay. Each condition consisted of 10 trials. If the subject chose the incorrect well on a given trial, the experimenter immediately pulled both wells away from the subject’s transport cage and revealed the food reward hidden in the unselected well. The total number of trials where the subject chose the correct well was recorded for each delay condition.

#### Food extraction puzzle

A small piece of clear plastic tubing containing half of a grape at its midpoint was placed inside the front of the subject’s transport cage. The subject was allowed to manipulate the tube in an attempt to extract the grape for a maximum of ten minutes. The subject had to solve the puzzle by extracting the grape using its tongue rather than its hands, which were too large to fit inside the tube. The time required by the subject to extract the food reward was measured for each trial. The subject was run in two consecutive ten-minute trials within one session of this task.

### Model Specification and Estimation

Although batteries of cognitive tests administered to humans show a clear dominant dimension, this dimension is not enough to capture the full correlational structure of the tests. The necessary additional dimensions are often called group factors, meant to capture the possible clustering effect of different cognitive abilities, perhaps linked to a single neuroanatomical region or circuit in the brain. In specifying a multiple-factor structure for our data, we grouped the tasks according to what we consider, together with other researchers, to be core cognitive processes. In particular, occluded reach, A-not-B, and reversal learning were all taken as indicators of executive inhibition of reflexive lower-level responses. Numerical discrimination and the food extraction puzzle were specified as indicators of a problem-solving factor. Object tracking and social tracking were specified as indicators of an “inspection” factor.

All replications of a given task were collapsed. Attempts to fit a confirmatory factor model to the resulting data with standard structural equation modeling software resulted in convergence failures from a wide range of starting values. This is perhaps unsurprising as our data departed in several ways from the ideal conditions for the fitting of standard factor models with maximum likelihood. These departures include small sample size (exacerbated by a high rate of missingness) and diversity across tasks of data form and distribution (non-normal continuous measurements, counts, binary outcomes).

To accommodate the distinctive features of our data, we employed a Bayesian latent variable model that has been successfully implemented in previous studies of primate cognitive performance [9], [19]; see Supplemental Protocol S1 for relevant notation and further details. This approach mitigates the limitations of small sample size and missing data by allowing estimates of a parameter such as a task’s non-*g* variance to borrow strength from estimates of related parameters (e.g., the corresponding variances of other tasks). Further, it handles the disparate data forms and distributions across tasks by converting them to the common format of ranks. Lastly, because our study, and the comparative analysis of the general factor across taxa make use of the same statistics, we can more directly compare our results.

In brief, a latent variable was invoked to underlie each task to account for the varying forms of the data, much as in the standard generalized linear model. For example, if a task produced dichotomous outcomes, then the model stipulated that a standing on the latent variable less than a certain threshold resulted in the lower rank for that animal; a standing on the latent variable higher than the threshold resulted in the higher rank. This notion generalizes in an obvious way to tasks producing more than two ranks. Three sources of variance were modeled for each task’s underlying latent variable: (1) a general factor affecting performance on all tasks, (2) a group factor affecting only a subset of the tasks, and (3) influences such as experimental error affecting that task alone. Our main interest is in estimating the proportion of each task’s latent variance attributable to the general factor. Recall that the *likelihood* is the probability that a certain configuration of model parameters will produce the observed data. In Bayesian statistical inference, the product of the likelihood and the *prior* probability of the configuration of model parameters is proportional to the desired *posterior* probability of the configuration. (The prior can be chosen in such a way that the marginal posterior probability distributions of important parameters are not sensitive to the prior’s precise specification.) Because the mathematical form of the posterior probability is not analytically tractable, values of the model parameters were sampled in proportion to their posterior probability according to a hybrid Gibbs-Metropolis algorithm that has been described elsewhere [25].

## Supporting Information

Supplemental Protocol S1 Complete description of the cognitive tasks and more complete description of the statistical modeling.
(0.07 MB DOC)

Figure S1 Plot of prior density overlaid on posterior histogram of important model parameter.
(0.57 MB TIF)

Figure S2 Plot of prior density overlaid on posterior histogram of important model parameter.
(0.58 MB TIF)

Table S1 Matrix of probabilities that given task has a higher loading on the general factor than any other task.
(0.04 MB DOC)

Table S2 Rank data of animal performance on cognitive tasks.
(0.00 MB DOC)

Table S3 Descriptive statistics of task performance.
(0.37 MB DOC)
