# Supplementary material for: General Intelligence in Another Primate: Individual Differences across Cognitive Task Performance in a New World Monkey (Saguinus oedipus)
Source: PLoS One. 2009 Jun 17;4(6):e5883. doi: 10.1371/journal.pone.0005883 (PMC2690653; doi:10.1371/journal.pone.0005883)
Supplement: Supplemental Protocol S1 — Complete description of the cognitive tasks and more complete description of the statistical modeling. (0.07 MB DOC) [file pone.0005883.s001.doc]

**Supplemental Protocol**

**Detailed Task Descriptions**

*Occluded reach.* This task measured the subject’s ability to inhibit an immediate

cognitive and motor response in order to access a desired food reward. The subject watched as

the experimenter placed a quarter of a piece of Froot Loop cereal in one of three positions behind a transparent Plexiglas barrier (10 x 10 cm) placed 15 cm in front of the subject’s cage. The subject was then able to reach through a long rectangular hole (20 x 4 cm) spanning the length of the transport cage door in order to access the Froot Loop. In the right and left conditions, the experimenter placed the Froot Loop at the edge of either side of the Plexiglas barrier, such that half of the Froot Loop was positioned behind the barrier, but the other half was exposed to the side of the barrier. For these conditions, the subject had to reach toward the sides of the barrier in order to access the partially exposed Froot Loop. In the center condition, the Froot Loop was located directly behind the center of the transparent barrier. For this condition, the subject could only access the Froot Loop by inhibiting a direct reach forward toward the Froot Loop, which would have resulted in collision with the barrier. Instead, the subject was required to reach around the side of the barrier to access the Froot Loop. The subject participated in five sessions of this task consisting of twelve trials each over five consecutive days. Each Froot Loop position condition was presented four times within a session, and condition order was counterbalanced across all twelve trials. The percentage of 20 total center condition trials in which the subject successfully reached around the barrier to access the Froot Loop was recorded across all five sessions.

*A not B.* This task measured the subject’s ability to inhibit a learned motor response.

Two opaque barriers (5 x 10 cm), A and B, were placed in front of the subject’s transport cage.

For all subjects, barrier A was on the left and barrier B was on the right. The subject watched as the experimenter placed a Froot Loop quarter behind barrier A. The subject was then allowed to reach through a long rectangular hole (20 x 4 cm) spanning the length of the transport cage door in order to reach behind either barrier A or B to access the food reward. Froot Loop quarters were placed behind barrier A for the first 5 trials. On trial 6, the subject watched the experimenter place the Froot Loop quarter behind barrier B instead. To access the Froot Loop quarter in this trial, the subject had to inhibit a learned association between a reach toward barrier A and the food reward. The subject’s success in choosing barrier B on trial 6 was recorded.

*Reversal learning.* In this task, the subject was required to learn an association between a

food reward and a particular colored glove and then subsequently to reverse this association.

The experimenter wore a green glove with a floral design on one hand and a solid-colored black

glove on the opposite hand. Glove position was counterbalanced by hand side (right or left) for

all trials for each session and phase of the task. All sessions and phases were administered on

consecutive days. Each subject’s performance was ranked independently for each separate phase. Subjects that passed a phase with a fewer number of sessions received a higher rank than those who required more sessions to pass. Average rank across all three phases was used to compute each subject’s overall performance rank for this task.

*Phase 1*. Phase 1 was a training phase in which the subject was familiarized with

the task paradigm. The experimenter placed an unconcealed food reward (¼ piece of a Froot

Loop) in one of her two open, gloved hands. After a two-second presentation of her hands 30 cm

in front of the subject’s transport cage, the experimenter moved her hands toward the cage to

allow the subject to make a choice. The subject made a choice by reaching a hand or mouth

through one of two holes (3 x 4 cm) in the transport cage door corresponding to the

experimenter’s right and left hands. This same presentation procedure was used for all

subsequent phases of the task. In phase 1, a choice was marked whenever the subject

successfully took the food from the experimenter’s hand. Subjects were randomly separated into

two groups, with one group always receiving the food reward in the green glove and the other

group always receiving the food reward in the black glove. Each session of phase 1 consisted of

twelve trials. A subject passed phase 1 by correctly choosing the glove containing the

unconcealed food reward on ten out of twelve trials within a session. If a subject failed to pass

phase 1 after seven sessions, that subject was aborted and the task was ended. The number of

sessions required to pass phase 1 was recorded.

*Phase 2.* In phase 2, the subject was required to learn an association between a

particular colored glove and a *concealed* food reward. Each session of phase 2 began with one

warm-up trial in which food was presented to the subject, unconcealed, in the same colored

glove as in phase 1. Following this warm-up trial were twelve experimental trials in which the

food reward was concealed inside one of the experimenter’s fists. For all experimental trials, the

food reward was again located in the same colored glove to which the subject had been originally

assigned in phase 1. This was done to reinforce a learned associated between the food reward and a particular colored glove. In phase 2, a choice was marked whenever the subject made

physical contact using either a hand or mouth with the experimenter’s closed fist. As soon as

contact was made, the experimenter opened both fists, allowing the subject to access the contents

of the hand that was selected. The opposite, non-selected hand was immediately retracted

outside of the subject’s reach. If the subject failed to make any choices on the first four experimental trials, intermediate “curled trials” were commenced for the remainder of the session. On these curled trials, the experimenter presented both closed fists to the subject, then slowly opened both fists outside of the subject’s reach to reveal the concealed food reward inside. Next, the experimenter reformed two fists and allowed the subject to make a choice. These trials were included to encourage subjects who did not understand that food was concealed inside the experimenter’s closed fists to make choices on subsequent trials. To pass phase 2, the subject was required to make ten out of twelve correct choices on non-curled, experimental trials on two consecutive sessions. If a subject failed to pass after seven sessions, that subject was aborted and the task was ended. However, if a subject succeeded on ten out of twelve experimental trials by the seventh session, that subject was given up to ten total sessions to pass phase 2. The number of sessions required to pass phase 2 was recorded.

*Phase 3.* In phase 3, the subject was required to reverse the initial food-color

association learned in phases 1 and 2. For this phase, no warm-up trial was included and the

color of the glove containing the food reward was immediately reversed. Thus, subjects

originally assigned to the green glove condition now received the concealed food reward in the

black glove and vice versa. Each session of phase 3 consisted of twelve experimental trials. In

order to pass phase 3, the subject was required to choose correctly on ten out of twelve trials on

one session. If a subject failed to pass after seven sessions, that subject was aborted and the task

was ended. The number of sessions required to pass phase 3 was recorded.

*Exploration.* In this task, novelty preference (dependent upon both inhibitory and

attentional processes) was assessed by measuring the subject’s motivation to explore a novel

open-field environment and to engage with novel stimuli. The subject’s behavior was monitored

inside a large, covered open-field box (3 x 1.5 x .3 meters) constructed of white, opaque plastic

panels. The box was covered by four transparent Plexiglas panels (75 x 38 cm). Black electrical

tape was used to mark four quadrants of equal dimension (75 x 38 cm) on the floor of the box.

This grid allowed for subject movement inside the box to be analyzed by quadrant.

Subjects participated in seven conditions of this task on consecutive days. For each condition, the subject was allowed to enter the open-field box through a door (30 x 30 cm) at one end of the box. If the subject failed to enter the open-field box within three seconds, a sliding panel inside the subject’s transport cage was pushed forward to force the subject out of its cage and into the open-field box. Once inside the open-field box, the box door was closed, preventing the subject from re-entering the transport cage. The subject was allowed to move about freely inside the box for the duration of five minutes. During this time, the experimenter retreated to the back wall of the experimental room and sat quietly on a stool.

For each of the seven task conditions, the subject encountered a different stimulus located directly in the center of the open-field box. Stimulus conditions were presented in the following order: baseline 1, woodchip, plastic cricket, leaves and branches, honey, robotic dog, and baseline 2. For both baseline conditions, no stimulus was present inside the open-field box. For the other five conditions, subjects were allowed to engage freely with the relevant stimulus. In the woodchip condition, a block of wood (45 x 10 x 20 cm) containing ten wells (10 cm deep) was positioned in the center of the open-field box. A colored stone was placed inside each and was covered with woodchips. The subject was able to dig through the woodchips in the wells to retrieve the colored stones. For the plastic cricket condition, the subject encountered a small 5 cm, stationary cricket toy. In the leaves and branches condition, a large patch of polyurethaned branches and fake leaves were arranged in a circular heap approximately 45 cm in diameter in the center of the open-field box. In the honey condition, the subject was allowed to lick at a petri dish containing a thin, 2 mm layer of honey. Finally, in the robotic dog condition, a red, plastic robotic dog approximately the size of a tamarin (25 x 12 cm) was placed in the center of the open-field box. The robotic dog was set to “random action” mode, such that it performed a number of pre-programmed behaviors—walking, rolling over, and sitting down—at random intervals throughout the trial.

The subject’s activity in each condition was video-recorded so that behavior could later be coded using the computer software program, iMovie. Several dependent measures were examined for each stimulus condition in order to collectively approximate the subject’s overall exploratory behavior and novelty preference. These dependent measures included time spent moving (versus stationary), time spent in physical contact with the stimulus, and time spent in each quadrant of open-field box. Time spent in the farthest quadrant of the open-field box relative to the point of entry was included as a measure of motivation to incur potential dangers associated with traveling a far distance from a familiar home base (the subject’s transport cage). For the robotic dog condition, an additional dependent measure—time spent in the same quadrant as the robotic dog—was collected. This served as a measure of the subject’s motivation to remain in close proximity to a potentially threatening, animate object. Each subject’s performance was ranked independently for each of the seven task conditions. Subjects that spent a greater length of time in contact with the stimulus, moving, or in proximity to the robotic dog received higher ranks those that spent less time performing these behaviors. Of the seven original stimulus conditions, the four with the greatest coefficients of variation in subject performance on the dependent measures were used to compute each subject’s overall performance rank for this task. Average rank was determined by scoring performance on each dependent measure for the baseline 1, woodchip, honey, and robotic dog stimulus conditions.

*Numerical discrimination.* In this task, the subject was required to choose the larger of

two food quantities given multiple, varying numerical contrasts. The subject watched as the

experimenter loaded two clear petri dishes with varying quantities of food (¼ Froot Loop pieces)

at a distance of 15 cm in front of the subject’s transport cage. For each trial, the experimenter loaded the petri dishes with pieces of food according to the following numerical contrasts: 1 v. 2, 1 v.3, 1 v. 4, 1 v. 5, 2 v.3, 2 v. 4, 3 v. 4., and 4 v. 5. Two forced choice control conditions— 1 v. piece of wood and 1 v. piece of plastic—were also included to ensure that the subject was engaged in the task and understood the experimental paradigm.

Next, the experimenter showed the subject the tray on which the two petri dishes were

mounted for three seconds before sliding the tray against the transport cage door. The subject

was then allowed to choose the petri dish containing the larger amount of food by reaching a

hand or mouth out from one of two small holes (3 x 4 cm) in the transport cage door that

corresponded to the right and left side petri dishes. A choice was marked as soon as the subject

extended a body part through either the left or right hole.

The subject was run in three sessions of this task consisting of ten trials each over three

consecutive days. Numerical contrast order was randomized for all ten trials within each session.

The larger food reward was also counterbalanced by side to control for potential subject side

biases. The larger food reward was never permitted to fall on the same side for more than two

consecutive trials. The proportion of total attempted trials correct (out of a maximum of 30 trials) in which the subject chose the larger food quantity was recorded.

*Targeted reach.* This task provided a measure of perceptual speed by measuring the

subject’s ability to grasp a moving food reward. A cable-tie glued to the top of the subject’s

transport cage was loaded with one half of a raisin on its bottom tip. The cable-tie and raisin

were raised to a 60-degree angle by the side of the subject’s cage and then released, causing the

raisin to swing in a pendular motion in front of the subject’s cage door. The subject was free to

reach its hands or mouth out through a small, centered, square hole (3.8 x 3.8 cm) in the transport

cage door in order to grasp the swinging raisin. If the subject failed to grasp the raisin within one minute of the pendulum coming to a standstill in front of the subject’s cage, the trial was aborted. This procedure was repeated for five trials in a single session. Each trial was video-recorded so that subject behavior could later be coded using the computer software program, iMovie. The length of time required for the subject to successfully grasp the raisin was measured for each trial. Each subject’s performance was ranked independently for each of the five trials. Subjects that successfully grasped the raisin in a short amount of time received higher ranks than those that took longer to do so. Average rank across all five trials was used to compute each subject’s overall performance rank for this task. Rank scores were used to take into account subjects’ relative improvement in performance across multiple trials. This task was designed to be a simple measure of basic processing speed and planning that required subjects to respond to visual information in order to capture a moving target.

*Object tracking.* This task measured the length of inspection time that a subject spent

tracking each of two different stimuli—a raisin (food object) and a metal screw (non-food

object). The experimenter presented each stimulus for two seconds at a distance of 5 cm from

the subject’s cage door, and then waived the stimulus in front of the subject according to a fixed

presentation pattern comprised of straight lines, diagonal lines, and figure 8’s. This same pattern was repeated three times for a total presentation time of approximately 33 seconds. Only one experimenter performed this task so that stimulus presentation speed and cadence would be consistent across all subjects. The subject was exposed to both stimulus conditions within a single session. Subjects were randomly divided into two groups such that half received the non-food condition first, and the other half received the food condition first. The total percentage of presentation time that the subject spent looking at the moving stimulus was measured for each condition. If the subject looked away and later resumed tracking the stimulus, these subsequent trackings were also included in the inspection time measure. This task was designed to be a simple test of basic attentional processes.

*Food extraction puzzle.* In this task, the subject was required to solve an extractive

foraging puzzle using a task-appropriate strategy. A small piece of clear plastic tubing (5 x 3

cm) containing one half of a grape directly inside its center was placed inside the front of the

subject’s transport cage. The subject was allowed to manipulate the tube in an attempt to extract the grape for a maximum of ten minutes. The subject was required to solve the puzzle by extracting the grape using its tongue rather than its hands, which were too large to fit inside the tube. The trial was ended as soon as the subject succeeded in extracting the grape. If the subject failed to extract the grape within ten minutes, the trial was aborted. The subject was run in two consecutive ten- minute trials within one session of this task. Each trial was video-recorded so that subject behavior could later be coded using the computer software program, iMovie. The length of time required by the subject to extract the food reward was measured for each trial. Each subject’s performance was ranked independently for both trials. Subjects that successfully extracted the grape in a short amount of time received higher ranks than those that took longer to do so. Average rank across both trials was used to compute each subject’s overall performance rank for this task. Rank scores were used to take into account subjects’ relative improvement in performance across both trials.

*Acoustic discrimination.* In this task, the subject’s rate of habituation to four different

biologically meaningful acoustic stimuli was measured. The subject was repeatedly exposed to

these acoustic stimuli played through a speaker located on a wall 1 meter behind and 1 meter

above the subject’s transport cage. The sound intensity for the speaker was 70e75 dB.

The four acoustic stimuli conditions all presented in a single session included a tamarin

alarm call, a goshawk alarm call, and the contacts calls of two familiar tamarin members of the

lab colony. Stimulus presentation order was counterbalanced for all subjects. Stimuli were

played when the subject was looking down and to the right inside the transport cage in order to

allow for a maximum orientation response. A response was defined as orienting up and toward

the speaker behind the transport cage during the stimulus exposure or within 2 seconds of its

termination. The subject was repeatedly exposed to each stimulus over a number of trials until a

specific habituation criterion was achieved. The time between calls was always between 10 and

60 seconds. The subject habituated to the stimulus when it failed to orient toward the speaker for

three consecutive stimulus exposures. If the subject failed to habituate after 50 exposures of a

given stimulus, that subject was aborted for the given stimulus condition. The number of

exposures required to habituate to each of the four stimulus conditions was measured. Each subject’s performance was ranked independently for each of the four stimulus conditions. Subjects that habituated more quickly to the acoustic stimulus received higher ranks than those that took longer to habituate. Average rank across all four conditions was used to compute each subject’s overall performance rank for this task.

*Social tracking.* This task measured the length of inspection time that a subject spent

tracking a social stimulus. The subject was positioned inside a transport cage that was adjacent

to a second transport cage containing a stooge animal. The transport cages were divided by an

opaque barrier preventing the two animals from viewing each other except through four

“peepholes” located in each corner of the opaque barrier. The subject was allowed to watch the stooge through the barrier’s peepholes while the stooge was engaged in a foraging task that involved digging for five ¼ Froot Loop pieces in a woodchip-filled trough attached to the side of the stooge’s cage. Though the subject could easily view the stooge, the positioning of the adjacent cages largely prevented the subject from viewing the trough. As a result, the majority of the subject’s inspection time spent looking through the peepholes involved tracking the stooge, rather than the food stimulus. The subject was allowed to look through the peepholes during a single 60-second trial. The trial was video-recorded so that subject behavior could later be coded using the computer software program, iMovie. The total length of inspection time that the subject spent tracking the stooge through any of the four peepholes was measured.

*Hidden reward retrieval*. This task measured the subject’s ability to remember the

location of a hidden reward following variable delay lengths. The subject watched an

experimenter bury a ¼ Froot Loop piece in one of two food wells filled with woodchips at a

distance of 15 cm from the subject’s transport cage. Food location was counterbalanced by right

or left well side for all ten trials in each session of the task. For each condition, a different delay length was imposed before the food wells were pushed against the subject’s transport cage to allow the subject to choose one well from which to retrieve the hidden food reward. A choice was marked when the subject reached its hand out of one of two small holes (3 x 4 cm) in the Plexiglas transport cage door, each corresponding to one of the two food wells. The subject was exposed to seven delay conditions on consecutive days in the following order: no delay, 5 second delay, 10 second delay, 15 second delay, 20 second delay, 25 second delay, and 30 second. If the subject chose the incorrect well, the experimenter immediately pulled both wells away from the subject’s transport cage and revealed the food reward hidden in the un-selected well. The total number of trials on which the subject chose the correct well containing the hidden food reward was recorded for each delay condition. In order to compare subjects’ rate of performance decline as longer time delays were imposed, subject performance was ranked for each delay condition separately. An average rank across all conditions was computed for each subject and used as the primary dependent measure for this task.

**Statistical Modeling and Analysis**

All replications of a given task were collapsed such that each task produced a single ranking of the participating subjects. Let *yij* denote the rank of the *i*th subject in the *j*th task, and let *g(j)* denote the group factor of which the *j*th task is an indicator. There are *I* total subjects ranked, *J* total experimental tasks, and *K* group factors (where *K* < *J*). We now invoke a latent variable *zij* to describe the capacity of the *i*th subject in the *j*th task. *yij* > *ykj* implies *zij* > *zkj* Note that smaller values of *y* and *z* indicate superior performance or capacity. We employ the model

*zij* = *θi* + *ηi,g(j)* + *εij*, (1)

where *θi* is the score of the *i*th subject on the general factor, *ηi,g(j)* is the score of the *i*th subject on a factor affecting all tasks conducted within group *g(j)*, and *εij* is an error term. This is in fact a very simple model. It says that an subject outranks another in a specific task only if the sum of the subject's score on the general factor, its score on the task's group factor, and an error term is smaller than the corresponding sum characterizing the second subject.

To establish a scale of measurement for the latent variables appearing in Equation (1), we follow Johnson et al. [25] and Deaner et al. [9] and assume that

*i* ~ N(0, 1),

*i,g(j)* ~ N(0, 1/*τg(j)*),

*εij ­*~ N(0, 1/*γj*), (2)

where N(*a, b*) refers to a normal distribution with mean *a* and variance *b*.

We next model the probability that two subjects *i* and *j* tie in their performance of a task by assuming that the probability of a tie is given by

*p­ij* = exp(–|*zi*+1,*j* – *zij*|/*κ*). (3)

The probability that subjects *i* and *j* do not tie on the specified task is 1 – *pij*. The negative exponential function decreases monotonically as its argument decreases. Thus, the probability of a tie decreases with increasing distance between *zi*+1,*j* and *zij*. How rapidly the probability of a tie decreases with this distance depends on the parameter *κ*, to which we give a uniform prior distribution on the positive real line.

Note that the prior distributions of the parameters given so far are not prejudiced in favor of a hypothesis invoking a general factor. While it is true that some variability in the *θi* is posited *a priori*, very small values of *ηi,g(j)* or *εij* will lead in the limit to a value of zero for the proportion of the variance in the latent variable underlying the task that is attributable to the general factor.

Each *γj* and *τg(j)* has its own prior distribution:

*γj* ~ Gamma(**1, **1/*μ*1),

*g(j)* ~ Gamma(**2, **2/*μ*2), (4)

where Gamma(*a*, *b*) refers to a gamma distribution with parameterization

Gamma(*x*|*a*, *b*) = [*ba/*(a)]*xa* – 1*e*–*bx*.

The prior distributions of each *γj* and *τg(j)* in turn depend on a third level of parameters:

*k* ~ Exp(*α*),

*k*~ Inv-Gamma(*β*, *δ*), (5)

for *k* = 1, 2, where Exp(*a*) refers to an exponential distribution with rate *a* and Inv-Gamma(*a*, *b*) to an inverse gamma distribution with parameterization

Inv-Gamma(*x*|*a*, *b*) = [*ba*/(a)]*x*–(*a*+1)*e*–*b*/x.

Following Johnson et al. [25], we take (*α*, *β*, *δ*) = (0.05, 0.65, 1.17) so as to represent a reasonable level of uncertainty in the final stage of the model hierarchy. The results are fairly insensitive to the precise choice of (*α*, *β*, *δ*).

Details regarding the Gibbs-Metropolis algorithm used to estimate this model can be found in Johnson et al. [25].

The posterior distribution of the parameters can be used to determine the probability that one task has a higher loading on the general factor than some other task. Supplemental Table S1 gives these probabilities.

In order to check the fit of our model, we examined the posterior histograms of *μ*2 and **2. These parameters are critical because they govern the distribution of the *γj*—the magnitudes of which determine the proportions of the task variances attributable to the general factor. The solid lines in Supplemental Figures S1 and S2 represent the prior densities of the parameters. If the prior and posterior distributions coincide very closely, then it is likely that the data contain little information about the parameters. On the other hand, if the prior and posterior distributions are extremely discordant, then the prior misspecification implies a poorly fitting model. As can be seen in Figures S1 and S2, neither problem is evident; while the prior and posterior distributions are not overly concordant, the prior distributions do appear to give adequate density to all plausible values of the parameters.
