# Supplementary figures and images for: General Intelligence in Another Primate: Individual Differences across Cognitive Task Performance in a New World Monkey (Saguinus oedipus)
Source: PLoS One. 2009 Jun 17;4(6):e5883. doi: 10.1371/journal.pone.0005883 (PMC2690653; doi:10.1371/journal.pone.0005883)

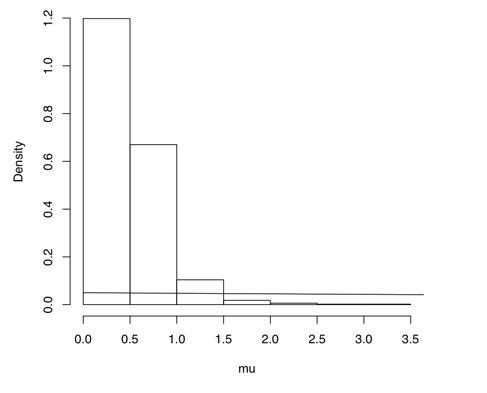

Supplement: Figure S1 — Plot of prior density overlaid on posterior histogram of important model parameter. (0.57 MB TIF) [file pone.0005883.s002.tif]

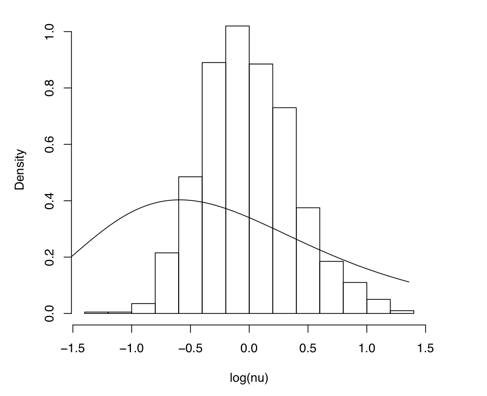

Supplement: Figure S2 — Plot of prior density overlaid on posterior histogram of important model parameter. (0.58 MB TIF) [file pone.0005883.s003.tif]
