# Supplementary material for: General Intelligence in Another Primate: Individual Differences across Cognitive Task Performance in a New World Monkey (Saguinus oedipus)
Source: PLoS One. 2009 Jun 17;4(6):e5883. doi: 10.1371/journal.pone.0005883 (PMC2690653; doi:10.1371/journal.pone.0005883)
Supplement: Table S1 — Matrix of probabilities that given task has a higher loading on the general factor than any other task. (0.04 MB DOC) [file pone.0005883.s004.doc]

*Posterior Probabilities of Relative Task Sensitivities As Indicators of the General Factor*

|  | (1) | (2) | (3) | (4) | (5) | (6) | (7) | (8) | (9) | (10) | (11) |
| --- | --- | --- | --- | --- | --- | --- | --- | --- | --- | --- | --- |
| (1) A-not-B |  | .27 | .19 | .14 | .09 | .07 | .04 | .04 | .03 | .02 | .02 |
| (2) occluded reach | .73 |  | .38 | .31 | .17 | .19 | .10 | .08 | .08 | .05 | .04 |
| (3) reversal learning | .81 | .62 |  | .44 | .27 | .30 | .16 | .14 | .13 | .09 | .08 |
| (4) food extraction | .86 | .69 | .56 |  | .31 | .31 | .20 | .17 | .16 | .09 | .09 |
| (5) object tracking | .92 | .83 | .73 | .69 |  | .50 | .37 | .30 | .30 | .22 | .19 |
| (6) num. discrim. | .93 | .81 | .70 | .69 | .50 |  | .37 | .31 | .30 | .21 | .22 |
| (7) acoustic discrim. | .96 | .90 | .84 | .80 | .63 | .63 |  | .42 | .43 | .30 | .28 |
| (8) exploration | .96 | .92 | .86 | .83 | .70 | .69 | .58 |  | .48 | .37 | .37 |
| (9) hidden reward | .97 | .92 | .87 | .84 | .70 | .70 | .57 | .52 |  | .39 | .39 |
| (10) social tracking | .99 | .95 | .91 | .91 | .78 | .79 | .70 | .63 | .61 |  | .51 |
| (11) targeted reach | .99 | .96 | .92 | .91 | .81 | .79 | .72 | .63 | .61 | .50 |  |
| *Note*. The *ij*th entry gives the posterior probability that task *j* has a higher loading on the general factor than task *i*. For example, the posterior probability that occluded reach has a higher loading on the general factor than task A-not-B is 0.27. | | | | | | | | | | | |
